# Mutation profile and molecular heterogeneity in mismatch repair deficient endometrial carcinoma

**DOI:** 10.3389/fonc.2025.1596879

**Published:** 2025-10-21

**Authors:** Yumeng Cai, Jing Wang, Zijuan Zhang, Pan Li, Jiuyuan Fang, Liang Cui, Sicong Xu, Yuhan Zhang, Junyi Pang, Yan You, Huanwen Wu, Zhiyong Liang

**Affiliations:** ^1^ Department of Pathology, State Key Laboratory of Complex Severe and Rare Disease, Molecular Pathology Research Center, Peking Union Medical College Hospital, Chinese Academy of Medical Sciences and Peking Union Medical College, Beijing, China; ^2^ Geneplus-Beijing Institute, Beijing, China

**Keywords:** endometrial carcinoma (EC), deficient DNA mismatch repair (dMMR), lynch syndrome, MLH1 promoter hypermethylation, lynch-like syndrome

## Abstract

Endometrial carcinoma (EC) with deficient DNA mismatch repair (dMMR) is a specific molecular entity with unique clinicopathological features. Herein, we depicted the mutation profile of dMMR ECs and explored the molecular heterogeneity among dMMR subgroups with different etiologies. Next-generation sequencing (NGS) based on a 1021-gene panel was applied to 74 dMMR ECs and 43 proficient DNA mismatch repair (pMMR) ECs. In addition, methylation-specific Polymerase Chain Reaction (PCR) was applied for accessing *MLH1* promoter hypermethylation (*MLH1* ^me+^) in dMMR cases. The mutation rates of *PTEN*, *ARID1A*, *KRAS*, and *MSH2* were significantly higher in dMMR group, while the *CTNNB1* and *MSH3* mutations were more commonly observed in pMMR group (p<0.05). Compared to pMMR ECs, dMMR ECs had significantly higher alteration frequencies in WNT, NOTCH, Cell Cycle and MMR, HRR and BER pathway (p<0.05). Remarkably, the interaction patterns within and across pathways were different between dMMR and pMMR groups. Intriguingly, no *CTNNB1* mutation were found in dMMR ECs, while half of the WNT-activated pMMR ECs were *CTNNB1* mutated, which were generally mutually exclusive with other WNT pathway key genes. The median tumor mutational burden (TMB) of dMMR ECs was significantly higher than pMMR ECs. However, ultra-high TMB value was related to pathogenic *POLE* mutation both in dMMR and pMMR ECs. As for dMMR subgroups (*MLH1* ^me+^, Lynch and Lynch-like), *KEAP1* and *FBXW7* mutations, which may have potential predictive effect of immunotherapy, were enriched in the Lynch subgroup. dMMR ECs has distinctive genomic profile with molecular heterogeneity, which may have potential prognostic and therapeutic implications.

## Introduction

Endometrial carcinoma (EC) is one of the most prevalent gynecological malignancies worldwide and represents the sixth and eighth leading cause of cancer-related death in the United States and China, respectively (1). In addition to histopathological classification, molecular characteristics have become another key dimension used to categorize ECs, providing additional prognostic and therapeutic information (2).

The Cancer Genome Atlas (TCGA) Research Network established the foundational molecular classification of EC, identifying four distinct subtypes including the microsatellite instability-high (MSI-H) subgroup, which has profound prognostic and therapeutic implications (3). Roughly 30% of ECs present with DNA mismatch repair deficiency (dMMR)/MSI-H disease, which constitute a molecular entity with unique clinicopathological features. In previous studies, dMMR status has been reported to correlate with multiple adverse clinicopathologic variables in ECs, namely higher tumor grade, presence of lymphovascular invasion, and later tumor stage (4). However, reports regarding the association between tumor MMR status and clinical outcome in EC patients have been conflicting (3–5), indicating the potential heterogeneity among dMMR ECs. Three main etiologically distinct subgroups have been identified in dMMR cancers: The Lynch subgroup (pathogenic/likely pathogenic germline mutations in any of *MLH1*, *MSH2*, *MSH6*, *PMS2*, *EPCAM*); the *MLH1*-hypermethylated group (*MLH1* promoter hypermethylation without *MLH1* germline mutations); and the Lynch-like subgroup (neither MMR gene germline mutations nor *MLH1* promoter hypermethylation) (6). Our previous studies have revealed notable differences in gene mutation profiles and signaling pathway interaction patterns among various dMMR subgroups in colorectal cancers (6, 7). In ECs, a recent study also suggested that *MLH1* hypermethylated tumors would display certain distinct molecular features compared to MMR germline mutated tumors (8). Nonetheless, the comprehensive spectrum of cancer driver genes and canonical signaling pathways alterations in dMMR ECs, with a detailed depiction of the molecular heterogeneity, remains to be fully illustrated. dMMR status is considered as an effective biomarker for immune checkpoint blockade (ICB) therapy in all solid tumors, including ECs, and typically associated with higher TMB, increased tumor-infiltrating lymphocytes, and upregulated compensatory PD-L1 expression (4, 9). However, tumor immunogenicity and response to ICB therapy might vary among dMMR ECs subgroups with different etiologies (10). Research into the molecular heterogeneity of dMMR ECs might provide more information for individualized clinical decision-making.

In the present study, a consecutive dMMR EC cohort were investigated using comprehensive genomic profiling in comparison with proficient DNA mismatch repair (pMMR) ECs. We focused on key genes and pathways involved in tumorigenesis and progression, and aimed to reveal the characteristic genetic profiles and molecular heterogeneity of dMMR ECs, which might facilitate future individualized therapy.

## Materials and methods

### Patients enrollment and clinicopathological characteristics

One hundred and seventeen patients with EC who underwent laparoscopic or laparotomic total hysterectomy and bilateral salpingooophorectomy with or without sentinel lymph node biopsy or systematic lymph node dissection at Peking Union Medical Collage Hospital (PUMCH) between November 2017 and February 2019 were enrolled in this retrospective study. The dMMR group (n = 74) comprised consecutive cases identified during the study period, whereas the pMMR group (n = 43) comprised randomly selected cases from the same period, intended to provide a representative sample for comparison. All patients did not receive anticancer treatment before surgery and the FIGO stage (the edition of 2023) was recorded according to postoperative pathological report. Detailed clinical and pathological characteristics including age, FIGO stage, tumor grade/histology, surgical procedure and follow-up dates of all patients were summarized in **Table 1**. All patients have signed consent for germline testing. The study was approved by the Institutional Review Board of PUMCH (approval number: S-K2006).

**Table 1:** Patient characteristics in 117 EC patients, including 74 dMMR and 43 pMMR patients.

Clinicopathologic charactristics	Total(n=117)	dMMR(n=74)	pMMR(n=43)	P
Age				0.198
Median(range)	52(33-85)	55(33-85)	46(33-67)	
<60	87(74.4%)	52(70.3%)	35(81.4%)	
≥60	30(25.6%)	22(29.7%)	8(18.6%)	
FIGO stage				**<0.001**
I	72(61.5%)	57(77.0%)	15(34.9%)	
II	11(9.4%)	2(2.7%)	9(20.9%)	
III	22(18.8%)	14(18.9%)	8(18.6%)	
IV	12(10.3%)	1(1.4%)	11(25.6%)	
Tumor grade/histology				**0.028**
Endometrioid				
Grade1	38(32.5%)	29(39.2%)	9(20.9%)	
Grade2	52(44.4%)	33(44.6%)	19(44.2%)	
Grade3	25(21.4%)	12(16.2%)	13(30.2%)	
Non-endometrioid	2(1.7%)	0	2(4.7%)	
Surgical procedure				0.067
Laparoscopic	104(88.9%)	69(93.2%)	35(81.4%)	
Laparotomic	11(9.4%)	5(6.7%)	8(18.6%)	
Lymphadenectomy				**<0.001**
Sentinel node biopsy	37(31.6%)	18(24.3%)	25(58.1%)	
Systemic lymph node dissection	9(7.7%)	4(5.4%)	5(11.6%)	
Not done	65(55.6%)	52(70.3%)	13(30.2%)	
Median follow-up (range), months	31 month(4-48)			
Recurrence/Metastasis	20(33.3%)	3(4.1%)	17(39.5%)	**PFS<0.001**
Death	3(2.6%)	1(1.4%)	2(4.7%)	OS=0.104

EC, Endometrial carcinoma; dMMR, deficient DNA mismatch repair; pMMR, proficient DNA mismatch repair. Values in bold represent p-values greater than 0.05.

### MMR IHC

Immunohistochemical (IHC) staining was performed on 4-μm formalin-fixed paraffin embedded tissue sections using a BOND-MAX/BOND-III autostainer (Leica, Germany) and the following antibodies (MLH1, clone OTI4H4; PMS2, clone OTI4BR; MSH2, clone OTIRB1R; MSH6, clone EP49) (Beijing Zhongshan Golden Bridge Biotechnology, China) according to the manufacturers’ recommendations.

Tumors with intact IHC staining of all four MMR proteins (MLH1, PMS2, MSH2, and MSH6) were identified as pMMR tumors. dMMR was defined as the complete loss of nuclear staining for one or more MMR proteins in tumor cells, with concurrent positive internal control staining in non-neoplastic cells (such as stromal cells and lymphocytes) on the same slide. Representative images of intact and lost nuclear staining for all four MMR proteins were provided in **Supplementary Figure S1**.

### Targeting sequencing

Targeting sequencing was performed using hybrid capture-based NGS as our previous research (6, 7). In brief, DNA was obtained from formalin-fixed paraffin-embedded (FFPE) tumors and patient-matched normal tissue respectively. Genomic DNA libraries were applied to a 1021-gene panel including whole exons, selected introns of 288 genes, and selected regions of 733 genes (**Supplementary Table S1**). Single-stranded DNA nanoball (DNB) were sequenced by using 2x100 bp paired-end reads on the Gene+Seq-2000 instrument (GenePlus-Beijing). All NGS quality control metrics were comprehensively summarized in **Supplementary Figure S2**. The following established quality thresholds were rigorously maintained across all analyzed samples and subsequently validated through graphical representation: 1) a minimum of 80% of bases achieving a Phred quality score (Q-score) of ≥30; 2) ≥95% of the target regions covered at a depth of more than 100x; 3) mapping rates exceeding 95%; and 4) on-target rates/capture efficiency achieving at least 50%. Additionally, to account for variations in capture efficiency, samples with an on-target rate between 30% and 50% were still retained if the average sequencing depth on target exceeded 300x. The NGS quality control graphic data is provided in **Supplementary Figure S2**. Genetic alternations, including single nucleotide variants (SNVs), small insertions and deletions (Indel), copy number variants (CNVs), and gene fusions/rearrangements, were compared with each paired normal sample to distinguish germline and somatic mutations. Sequencing data were analyzed by BWA (11) (version 0.7.12-r1039). SNVs and small Indels were identified by Mu Tect2 (12) (version 4.1.8.1). Somatic mutations were identified by a VAF ≥ 1% and at least 5 high-quality reads (Phred score ≥30, mapping quality ≥30, and without paired-end reads bias). Gene mutations were annotated using ANNOVAR software (13). CONTRA software (14) was used to detect CNVs and BreakDancer software was used to detect cancer-associated gene fusion (15).

To calculate TMB, the number of somatic, coding, nonsynonymous single nucleotide variants, and insertions and deletions mutations per megabase (Muts/Mb) of genome examined was defined. TMB levels were divided into two categories: TMB-L (tumor mutation burden-low, 1–9 Muts/Mb) and TMB-H (tumor mutation burden-high, ≥ 10 Muts/Mb). Ultra-high TMB cases were defined as those with TMB≥100 Muts/Mb.

### Signal pathways, key genes, and determination of mutational significance

Ten canonical signaling pathways have been identified as frequently genetically altered in cancers, including the Cell Cycle pathway, Hippo pathway, MYC pathway, NOTCH pathway, NRF2 pathway, PI3K/Akt pathway, RTK-RAS pathway, TGF-β pathway, TP53 pathway, and WNT pathway, as determined by the Cancer Genome Atlas (TCGA) analysis (16). Within our 1021-gene panel, sixty genes were assigned to pathways based on a combined revision of pathway analyses in previous literature and expert opinion (17–26). Fifty-four genes have been identified as DNA damage response (DDR)-related genes in previous studies and our 1021-gene panel (27–29), and were assigned to eight DDR pathways: MMR, homologous recombination repair (HRR), Fanconi anemia (FA), base excision repair (BER), checkpoint factor (CPF), nucleotide excision repair (NER), nonhomologous end joining (NHEJ), and translesion DNA synthesis (**Supplementary Table S2**). Critically, to accurately reflect the biological reality that many DDR genes operate in multiple pathways, we assigned several core genes to all pathways in which they play a definitive role (e.g., *POLD1* in *BER* and *MMR*; *ATM* in *CPF* and *HRR*; *BLM*, *BRCA1*, *BRCA2*, *BRIP1*, *PALB2*, *RAD51* and *RAD51C* in FA and HRR; *RAD50* and *MRE11A* in HRR and NHEJ). Consequently, a mutation in one of these genes contributes to the alteration count for each of its assigned pathways.

Specific recurrent missense mutations, i.e., hotspot mutations, amplifications, or fusions of oncogenes were classified as activating events. Mutations of oncogenes were filtered according to the related documentation in the Catalog of Somatic Mutations in Cancer (COSMIC) (30) and OncoKB annotation (31). For tumor suppressor genes, all loss-of-function mutations, including nonsense mutations, frame-shifting mutations, and canonical splice sites mutations were part of an inactivation event and defined as “predicted deleterious” mutations. Missense mutations were considered deleterious when identified in two or more of the following *in silico* functional analysis algorithms: predication score 0.0–0.05 in SIFT (sorting intolerant from tolerant) (32), “possibly damaging” or “probably damaging” in polymorphism phenotyping -2 (Polyphen2) (33), or “medium” or “high” in MutationAssessor (34). For missense mutations in the DDR pathway, the functional effects were manually reviewed in the COSMIC, recurrent hotspot mutations (35) and PubMed searches, as previously described by Iyer et al. (36) and Teo et al (37).

### *MLH1* promoter hypermethylation analysis

*MLH1* promoter hypermethylation was detected in cases with absent *MLH1* expression and lack of MMR germline mutations. The detection was performed using PCR as previously described (7).

### Definition of dMMR EC subgroups

dMMR ECs were categorized into three subgroups according to different underlying mechanisms: the Lynch subgroup is defined as cases harboring pathogenic/likely pathogenic germline mutations in any of the MMR genes (*MLH1*, *MSH2*, *MSH6*, *PMS2*, *EPCAM*); the *MLH1*-hypermethylated subgroup is defined as cases with loss of MLH1/PMS2 expression exhibiting *MLH1* promoter hypermethylation without *MLH1* germline mutations; and the Lynch-like subgroup consisted of cases with neither MMR gene germline mutations nor *MLH1* promoter hypermethylation.

### Statistical analyses

For all analyses comparing mutation frequencies between groups, the curated list of functionally altered genes which derived from the ANNOVAR-annotated variants after applying the functional filtering criteria described above was first compiled. This compiled data was then stratified by MMR status or *MLH1* methylation subgroup for statistical comparison and visualization.

Categorical variables were expressed as percentages and were analyzed using Fisher’s exact test. For genomic alterations, a “per-patient, per-gene” method was used for the oncoprint and mutation frequency plot, where each gene was scored in a binary manner (altered or wild-type) for each patient, irrespective of the number of mutations within that gene, thus representing the prevalence of alterations. The Wilcoxon rank-sum test was used for comparisons between two groups of continuous variables, and the Kruskal-Wallis rank sum test was applied for comparisons across three or more groups of continuous variables. For the primary group comparisons in the study (dMMR *vs*. pMMR), the false discovery rate (FDR) correction was applied. In contrast, adjustment for multiple testing was not performed for exploratory subgroup analyses with limited sample sizes, such as comparisons among the three dMMR subgroups or between high-TMB and low-TMB subgroups within the pMMR cohort, in order to minimize the risk of overlooking potential biologically meaningful findings in these hypothesis-generating investigations.

Overall survival (OS) was defined as the time from the date of primary surgery to the date of death from any cause. For patients who were still alive at the last follow-up, their data were censored on the last known date of contact (March 20, 2021). Progression-free survival (PFS) was defined as the time from the date of primary surgery to the date of first documented disease progression, recurrence, metastasis, or death from any cause, whichever occurred first. Patients who were event-free at the last follow-up were censored on the date of their last disease assessment (March 20, 2021). The Kaplan-Meier method was used to estimate survival curves for different patient subgroups. Differences between survival curves were compared using the log-rank test. Analyses were performed with R software (version 4.4.1) software. All tests were two-sided, and a p-value <0.05 was considered statistically significant.

## Results

### The clinical and pathological characteristics of dMMR and pMMR patients

A total of 117 EC patients were included, comprising 74 patients with dMMR and 43 patients with pMMR. The clinical and pathological characteristics of these patients were summarized in **Table 1**. The median age of the entire cohort was 52 years (range: 33–85 years). The majority of patients in both groups were under 60 years of age (70.3% in dMMR *vs* 81.4% in pMMR). Significant differences were observed in FIGO stage distribution between the two groups (p<0.001). The dMMR group showed a predominance of early-stage disease, with 77.0% of patients presenting with stage I disease, compared to only 34.9% in the pMMR group. Conversely, advanced stage disease was substantially more common in the pMMR group (25.6%) than in the dMMR group (1.4%). Histopathological analysis revealed differences in tumor grade and histology between the groups (p=0.028). The dMMR group contained a higher proportion of grade 1 endometrioid carcinomas (39.2% *vs* 20.9%), while the pMMR group had more grade 3 tumors (30.2% *vs* 16.2%) and non-endometrioid histologies (4.7% *vs* 0%). Regarding surgical management, laparoscopic procedure was more frequently performed in dMMR patients (93.2%) compared to pMMR patients (81.4%), though this difference approached but did not reach statistical significance (p=0.067). Significant differences were noted in lymph node assessment strategies (p<0.001), with sentinel node biopsy being more common in pMMR patients (58.1% *vs* 24.3%), while no lymphadenectomy was more frequently performed in dMMR patients (70.3% *vs* 30.2%).

With a median follow-up of 31 months (range: 4–48 months), significant differences emerged in clinical outcomes. The recurrence/metastasis rate was substantially higher in pMMR patients (39.5%) compared to dMMR patients (4.1%), corresponding to a significant difference in PFS (p<0.001) (**Supplementary Figure S3A**). However, no significant difference was observed in OS p=0.104) between the two groups, with death occurring in 1.4% of dMMR patients and 4.7% of pMMR patients (**Supplementary Figure S3B**).

### Gene mutations in dMMR and pMMR ECs

**Figure 1:**
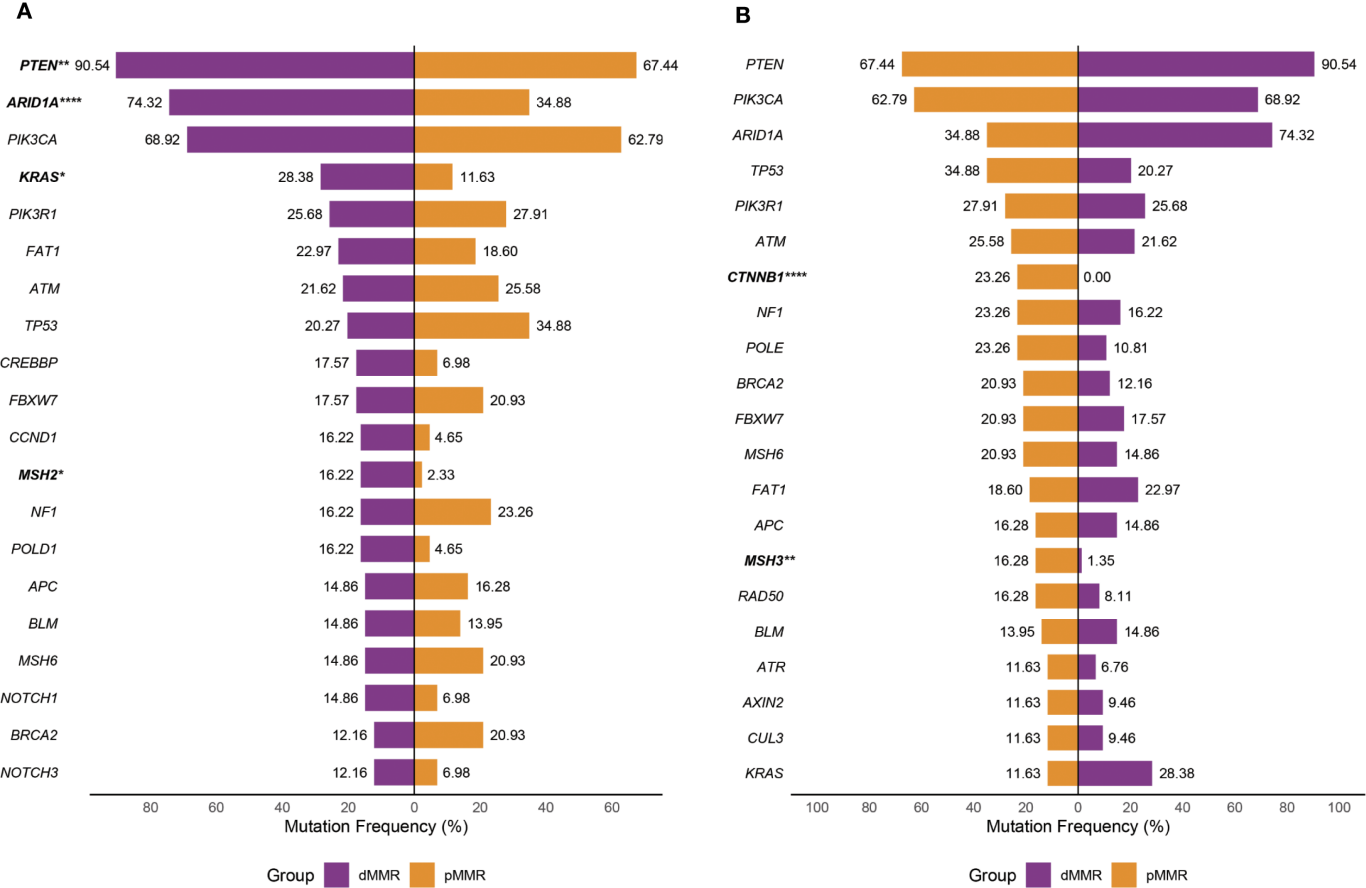
Mutation profile of recurrently mutated genes in the dMMR and pMMR ECs. Each gene is counted once per patient, irrespective of the number of mutations within that gene. **(A)** Prevalence of most frequently mutated genes in the dMMR ECs compared with that in the pMMR ECs. **(B)** Prevalence of the most frequently mutated genes in the pMMR ECs compared with that in the dMMR ECs. dMMR deficient mismatch repair, pMMR proficient mismatch repair, EC endometrial carcinoma, Asterisk (*) significant difference in mutation prevalence (Fisher’s exact test, *p < 0.05, **p < 0.01, ****p < 0.0001, FDR-corrected).

Of the key genes involved in ten canonical tumor-related pathways and DDR pathways, *PTEN*, *ARID1A*, and *PIK3CA* had the highest mutation frequency in both dMMR (90.5%, 74.3%, 68.9%) and pMMR (67.4%, 34.9%, 62.8%) ECs, followed by *KRAS* (28.4%), *PIK3R1* (25.7%), *FAT1* (23.0%), *ATM* (21.6%), *TP53* (20.3%), *CREBBP* (17.6%), and *FBXW7* (17.6%) in dMMR cohort, and *TP53* (34.9%), *PIK3R1* (27.9%), *ATM* (25.6%), *CTNNB1* (23.3%), *NF1* (23.3%), *POLE* (23.3%), and *FBXW7* (20.9%) in pMMR cohort. In comparison with those in the pMMR group, the mutation rates of *PTEN*, *ARID1A*, *KRAS*, and *MSH2* were significantly higher in the dMMR group (90.5% *vs*. 67.4%, p<0.05; 74.3% *vs*. 34.9%, p<0.05; 28.4% *vs*. 11.6%, p<0.05; 16.2% *vs*. 2.3%, p<0.05), while the *CTNNB1* and *MSH3* mutations were significantly higher in the pMMR group (0% *vs*. 23.3%, p<0.05; 1.4% *vs*. 16.3%, p<0.05) (**Figure 1**). We also noted that alterations causing functional impairment of tumor suppressor gene *ARID1A* were mostly frameshift events affecting homopolymer sequences in dMMR group as opposed to pMMR group (65/87, 74.7% *vs*. 4/26, 15.4%, p<0.05).

### Signaling pathway alternations in dMMR and pMMR ECs

**Figure 2:**
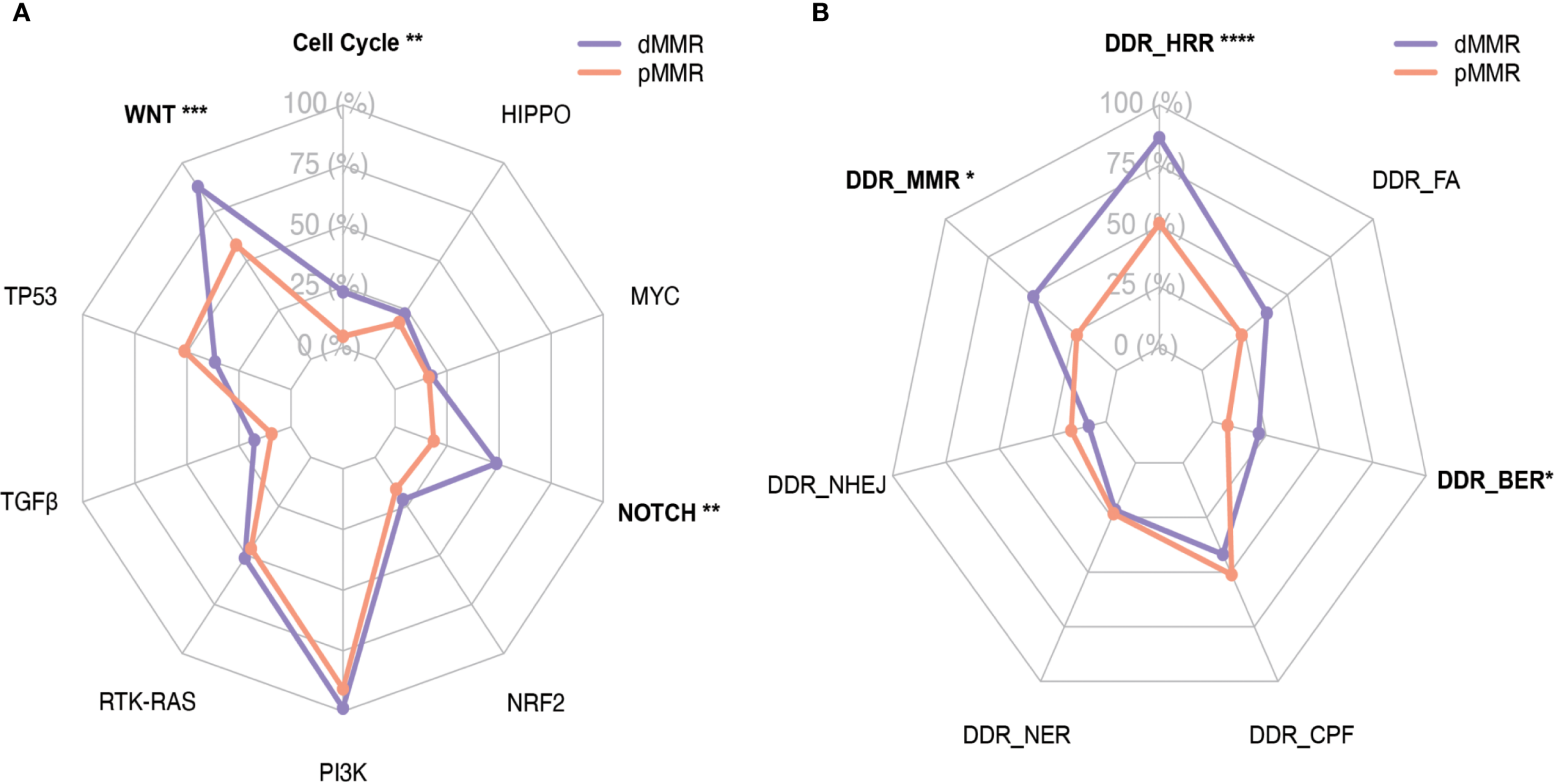
Distribution of WNT, RTK-RAS, PI3K, TGF-β, Cell Cycle, Hippo, NRF2, NOTCH, MYC and TP53 pathways and DDR pathways with respect to their mutation frequency in the dMMR and pMMR ECs. **(A)** Distribution of WNT (9 genes), RTK-RAS (8 genes), PI3K (7 genes), TGF-β (8 genes), Cell Cycle (7 genes), Hippo (5 genes), NRF2 (3 genes), NOTCH (6 genes), MYC (3 genes) and TP53 (4 genes) pathways. **(B)** Distribution of DDR pathways, inculding MMR (10 genes), HRR (25 genes), FA (15 genes), BER (6 genes), NHEJ (3 genes), NER (7 genes) and CPF (6 genes); dMMR deficient mismatch repair, pMMR proficient mismatch repair, EC endometrial carcinoma, DDR DNA damage response, MMR mismatch repair, HRR homologous recombination repair, FA Fanconi anemia, BER base excision repair,CPFs checkpoint factors, NER nucleotide excision repair, NHEJ nonhomologous end joining, and TLS translesion synthesis. Asterisk (*) significant difference in mutational prevalence (Fisher’s exact test, *p < 0.05, **p < 0.01, ***p < 0.001, FDR-corrected).

Pathway alteration analysis revealed distinct patterns between dMMR and pMMR ECs. The dMMR group showed significantly higher alteration frequencies in NOTCH (48.6% *vs* 18.6%, p=0.007), WNT (87.8% *vs* 58.1%, p=0.005), and Cell Cycle pathways (23.0% *vs* 4.7%, p=0.03) compared to pMMR, while exhibiting lower TP53 pathway defects (36.5% *vs* 51.2%, p=0.35). Both groups demonstrated high alteration rates in PI3K (dMMR 100%, pMMR 90.7%) pathway, but low frequencies in Hippo, NRF2, TGF-β and MYC pathways (**Figure 2A**).

Regarding DDR pathways, all dMMR tumors (100%) showed at least one alteration versus only 44.2% of pMMR tumors (p<0.001). The dMMR group particularly displayed elevated MMR (48.6% *vs* 23.3%, p<0.05), HRR (86.5% *vs* 51.2%, p<0.001) and BER (21.6% *vs* 7.0%, p<0.05) pathway alterations. No significant differences were observed in FA, CPF, NER or NHEJ pathways between groups (**Figure 2B**). Given the constraint of our gene panel, which included only three genes (*MRE11A*, *PRKDC* and *RAD50*) for the NHEJ pathway and one gene (*POLQ*) for TLS pathway, the alteration frequency we report is likely an underestimate, and these findings should be interpreted with caution.

### Mutual relationships among key genes within canonical pathways in dMMR and pMMR ECs

We then depicted the co-occurring and mutually exclusive relationship among mutations affecting key genes involved in ten canonical signaling pathways.

PI3K, NOTCH, and TP53 pathways often had multiple alterations per tumor sample. Within the PI3K pathway, *PTEN* mutations were frequently accompanied by *PIK3CA* and/or *PIK3R1* mutations in both dMMR and pMMR groups (90.5% and 67.4%, respectively). Co-alteration of *PTEN*, *PIK3CA*, and *PIK3R1*, the three key PI3K signaling genes, were found in 9 out of 74 (12.2%) dMMR tumors and 7 out of 43 (16.3%) pMMR tumors, respectively. On the contrary, the RTK-RAS pathway contained predominantly mutually exclusively altered genes. *KRAS* was the most commonly mutated RTK-RAS signaling gene in both dMMR and pMMR groups and exhibited an almost perfect mutually exclusive pattern with other members in the RTK-RAS pathway. The only exceptions were the co-occurrence of non-canonical *KRAS* mutations (A59T, A146V) and *NF1* deleterious mutations observed in four dMMR tumors.

The alteration spectrum of the WNT signaling pathway displayed noticeable differences between dMMR and pMMR groups. All WNT-activated dMMR tumors were *CTNNB1* wild-type, and the majority of them (16/24, 66.6%) displayed dysfunctional mutations in only one other key WNT signaling genes, including *APC*, *RNF43*, *AXIN1*, *AXIN2*, and *TCF7L2*. On the other hand, half of the WNT-activated pMMR tumors (10/18, 55.6%) were *CTNNB1* mutated, which were generally mutually exclusive with alterations in other WNT pathway components. The remaining eight WNT-activated pMMR tumors showed high frequency (6/8, 75%) of co-alterations among key WNT pathway genes other than *CTNNB1*.

**Figure 3:**
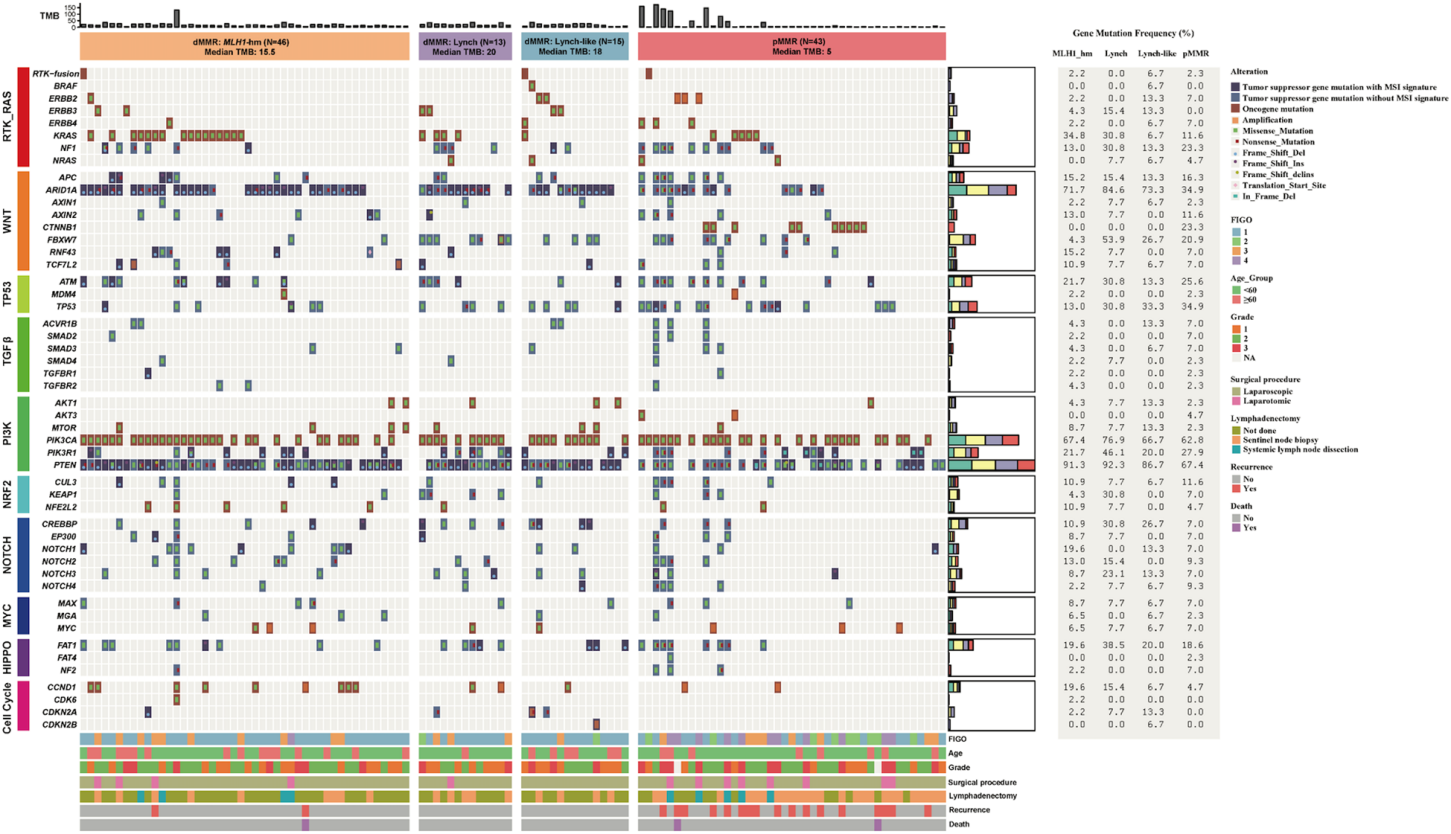
Mutation profile of key genes in the dMMR and pMMR ECs. The alteration profile includes single nucleotide variants (SNVs), small insertions and deletions (indels), and copy number variations (CNVs). Each gene is counted once per patient, irrespective of the number of mutations within that gene. Columns represent individual cases sorted by MMR status and dMMR subgroups. Tracks indicate WNT, RTK-RAS, PI3K, TGF-β, Cell Cycle, Hippo, NRF2, NOTCH, MYC and TP53 pathway gene mutations. Individual genes are listed in rows. dMMR deficient mismatch repair, pMMR proficient mismatch repair, EC endometrial carcinoma, MSI microsatellite stability, *MLH1*-hm hypermethylated *MLH1* promoter, Lynch Lynch syndrome-associated, Lynch-like Lynch-like syndrome-associated, TMB Tumor mutation burden, Del Deletion, Ins Insertion, delins deletion-insertion.

Mutations in key genes of the signaling pathways were shown in **Figure 3**.

### Mutual relationships between signaling pathways in dMMR and pMMR ECs

**Figure 4:**
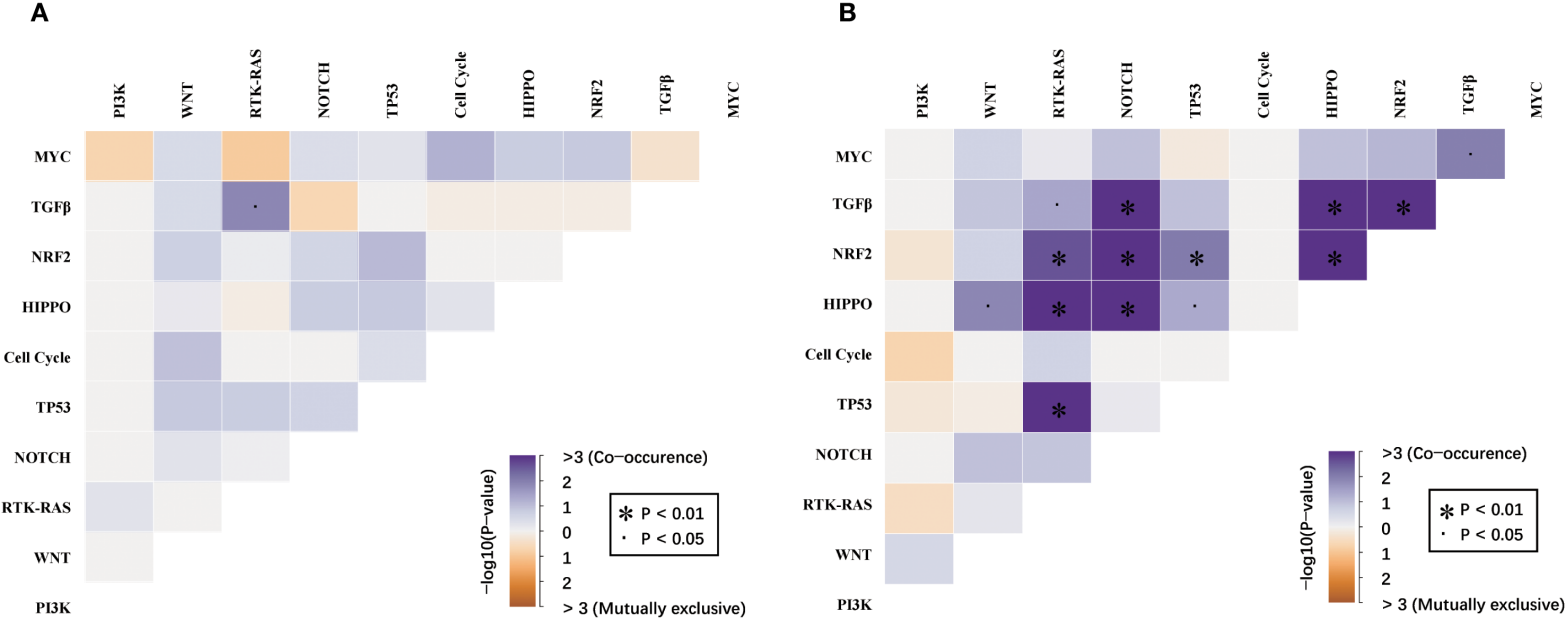
Mutual relationships among the WNT, RTK-RAS, PI3K, TGF-β, Cell Cycle, Hippo, NRF2, NOTCH, MYC and TP53 pathways between **(A)** dMMR and **(B)** pMMR EC. dMMR deficient mismatch repair, pMMR proficient mismatch repair, EC endometrial carcinoma. Co-occurrence and mutual exclusivity were identified using the Fisher’s exact test. The significance level is encoded in color representing −log10 (Fisher’s exact test,.p < 0.05, *p < 0.01, FDR-corrected).

The mutual relationship among the canonical signaling pathways differed remarkably between dMMR and pMMR ECs (**Figure 4**). In the dMMR group, the only significant mutually exclusive relationship was found between TGF-β and RTK-RAS pathway (p<0.05). No significant co-occurrence pattern among ten canonical pathways was observed. On the contrary, in the pMMR group, we identified numerous co-existence relationships within TGF-β, NOTCH, WNT, RTK-RAS, HIPPO, and NRF2 pathways (p<0.05). Despite ranking the most commonly altered pathway, PI3K was not significantly concurrently altered with any other pathways in pMMR ECs.

### Tumor mutational burden level in dMMR and pMMR ECs, and comparison of clinicopathological features by TMB status in pMMR tumors

**Figure 5:**
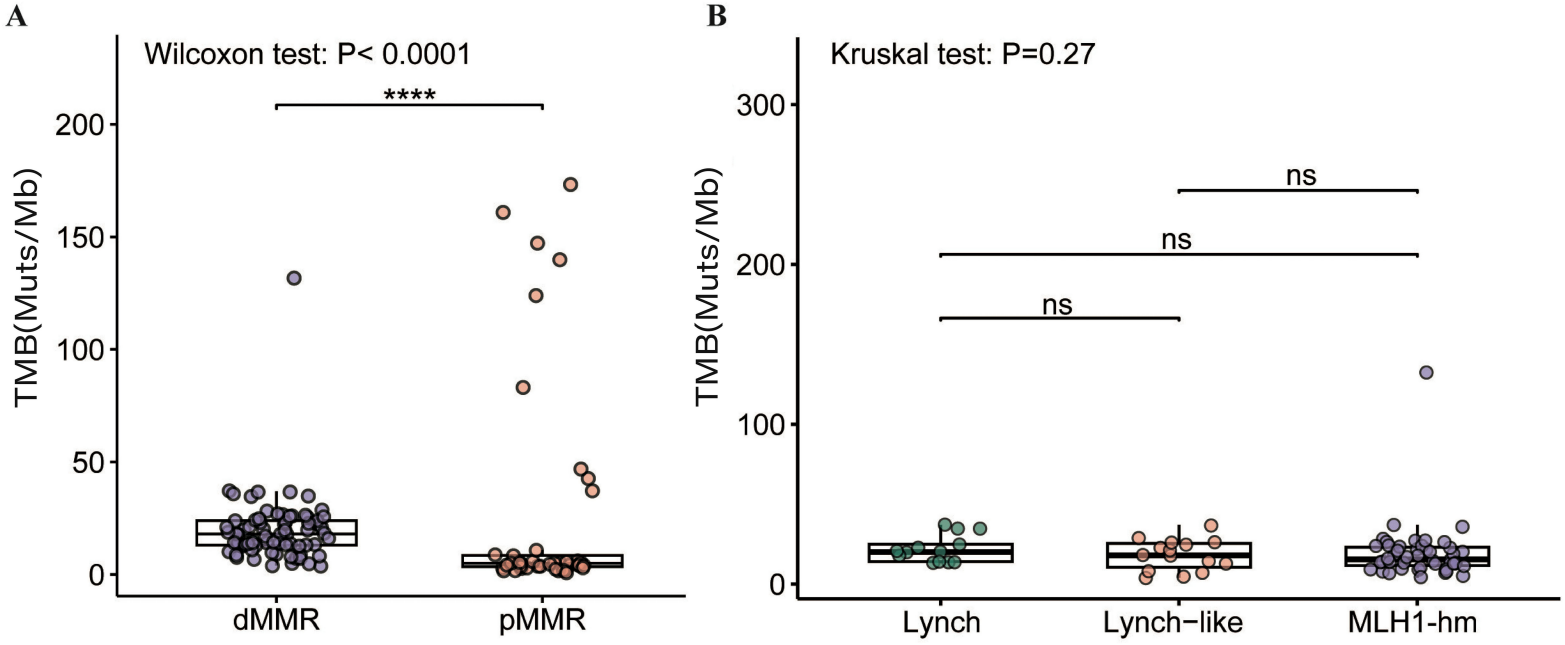
Comparison of TMB in the **(A)** dMMR EC and pMMR EC and **(B)** dMMR subgroups. dMMR deficient mismatch repair, pMMR proficient mismatch repair, EC endometrial carcinoma. *MLH1*-hm hypermethylated *MLH1* promoter, Lynch Lynch syndrome-associated, Lynch-like Lynch-like syndrome-associated, TMB tumor mutation burden, Muts/Mb mutations per megabase, ns no significance. Asterisk (*) significant difference in mutational prevalence (Fisher’s exact test, ****p < 0.0001, FDR-corrected).

As shown in **Figure 5A**, the median TMB of 74 dMMR tumors was significantly higher than that of 43 pMMR tumors (18 mut/Mb *vs*. 5 mut/Mb, p<0.0001). Of note, the highest TMB value (132 mut/Mb) was observed in only one dMMR tumor harboring somatic inactivating mutations in the exonuclease domain of *POLE* (S459F). Likewise, in the pMMR group, high TMB levels (≥10 mut/Mb) were generally found in tumors with deleterious *POLE* exo-domain mutations (P286R, S297F, F367S, V411L), with all nine high-TMB tumors harboring such alterations and TMB values ranging from 37 to 173 mut/Mb.

We next sought to determine if this genetically distinct subset of pMMR tumors with high TMB was associated with different clinical outcomes. The clinicopathological characteristics of the nine high-TMB pMMR patients were compared to the remaining pMMR cohort (n=34) (**Supplementary Table S3**). No significant differences were observed between the groups in age, histology, tumor grade, FIGO stage, surgical procedure, or lymphadenectomy status (all p > 0.05). However, the high-TMB group showed a trend toward lower recurrence/metastasis rate (22.2% *vs*. 44.1%) and mortality (0% *vs*. 5.9%). However, there were no significant difference in PFS (p=0.363) and OS (p=0.999) (**Supplementary Figure S4**).

### Subgroup analysis of dMMR and pMMR ECs

To address potential confounding effects from the uneven distribution of tumor stages between the dMMR and pMMR groups, we performed stratified analyses by grouping patients into early-stage (FIGO I-II) and late-stage (FIGO III-IV) cohorts. This confirmed that the key genomic differences between dMMR and pMMR tumors were consistent across disease stages. Specifically, within the early-stage cohort, dMMR tumors (n=59) exhibited a significantly higher TMB compared to pMMR tumors (n=24) (median TMB: 15 mut/Mb *vs* 4.5 mut/Mb, p<0.001). These differences remained robust and significant in the late-stage cohort (median TMB: 23 mut/Mb *vs* 5 mut/Mb, p<0.01) (**Supplementary Figure S5**).

For pathway analysis, the early-stage dMMR tumors (n=59) exhibited higher alteration frequencies in the NOTCH (45.8% *vs* 16.7%, p<0.05), WNT (86.4% *vs* 66.7%, p=0.062), Cell Cycle (23.7% *vs* 4.2%, p=0.056), DDR-HRR (84.7% *vs* 50%, p<0.01) and DDR-MMR (44.1% *vs* 20.8%, p=0.078) pathways compared to pMMR tumors (n=24). These differences remained robust and significant in the late-stage cohort, as NOTCH (60.0% *vs* 21.1%, p<0.05), WNT (93.3% *vs* 47.4%, p<0.01), DDR-HRR (93.3% *vs* 52.6%, p<0.05), DDR-MMR (66.7% *vs* 26.3%, p<0.05) and DDR-BER pathway (33.3% *vs* 0%, p<0.05) (**Supplementary Figure S6**). These findings indicate that the molecular characteristics we observed are intrinsically linked to MMR status, independent of tumor stage.

### Comparison of the clinical pathological features, TMB levels and mutation profile among ECs with different dMMR etiologies

The 74 dMMR ECs were categorized into three subgroups according to different underlying mechanisms: the Lynch subgroup (13/74, 17.6%); the *MLH1*-hypermethylated group (46/74, 62.2%); and the Lynch-like subgroup(15/74, 20.3%).

No significant differences were observed among these subgroups in any of the clinical pathological features, including age distribution (p=0.138), FIGO stage (p=0.279), tumor grade/histology (p=0.513), surgical approach (p=0.664), or lymph node management strategy (p=0.544). With a median follow-up of 35 months (range: 4–48 months), recurrence/metastasis events were exclusively documented in the *MLH1* ^me+^ subgroup (3 cases, 6.5%), while no such events were observed in either the Lynch or Lynch-like subgroups. Similarly, mortality was limited to one case (2.2%) in the *MLH1* ^me+^ subgroup (**Supplementary Table S4**). These differences in PFS (p=0.238) and OS (p=0.737) did not reach statistical significance among the three dMMR subgroups (**Supplementary Figure S7**).

No significant difference in TMB was observed among the dMMR subgroups. The median TMB was 20.0 mt/Mb in the Lynch subgroup, 18.0 mt/Mb in the Lynch-like subgroup, and 15.5 mt/Mb in the *MLH1*-hypermethylated subgroup (p > 0.05) (**Figure 5B**).

Mutation profile of key genes varied by dMMR etiologies. The proportion of cases with alterations in *KEAP1* (4/13, 30.8%) and *FBXW7* (7/13, 53.8%) were significantly enriched in Lynch subgroup (p<0.05). Conversely, *MSH2* (8/15, 53.3%) were significantly enriched in Lynch-like subgroup (p<0.0001). These findings, while statistically significant, are based on small sample sizes and warrant validation in larger cohorts.

**Figure 6:**
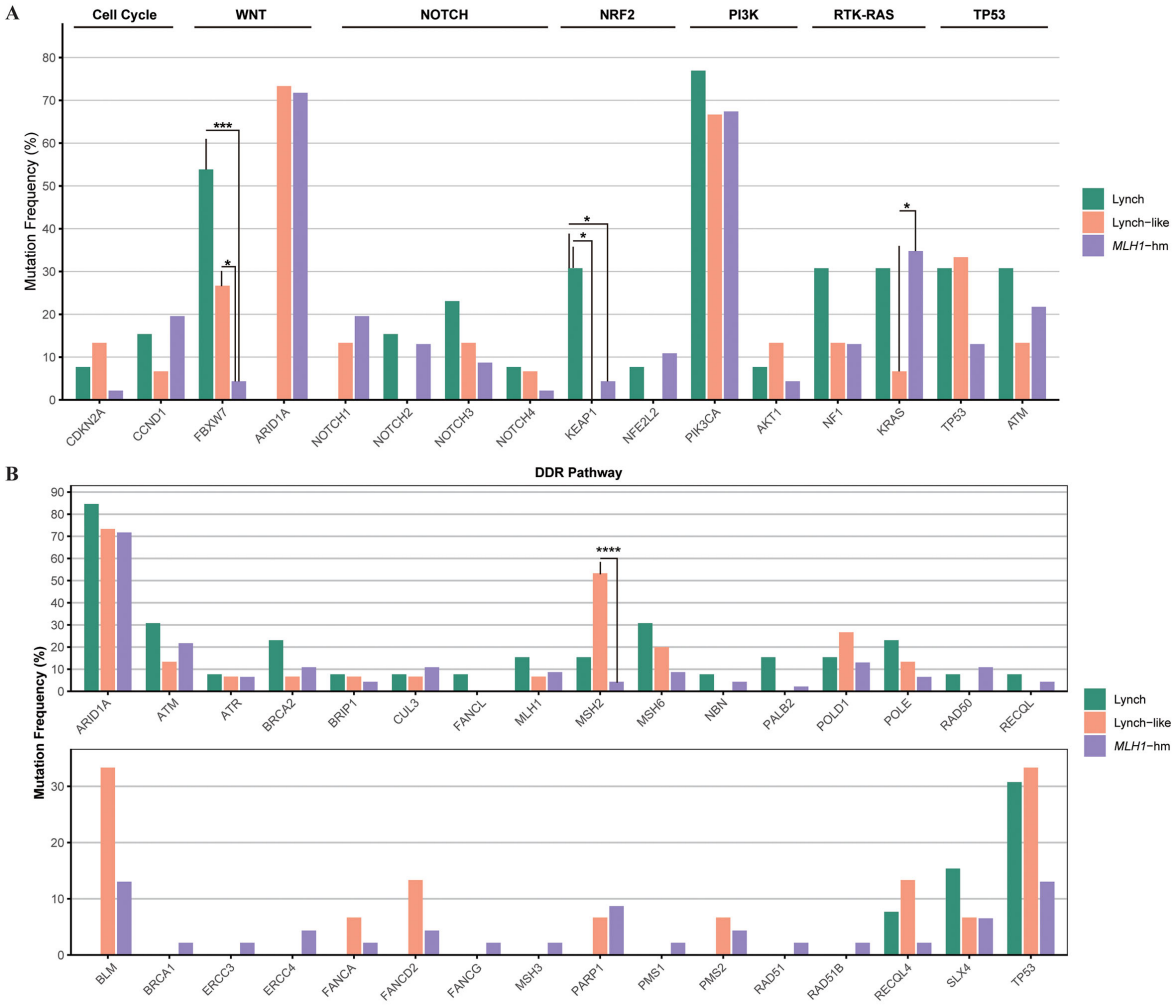
Comparison of the proportion of cases with alterations in selected genes across dMMR EC subgroups. **(A)** Mutational landscape of canonical signaling pathways. **(B)** Mutational landscape of DNA damage response (DDR) pathways. The bar height represents the percentage of patients within each subgroup harboring a mutation in the corresponding gene. dMMR deficient mismatch repair; EC endometrial carcinoma; MLH1-hm hypermethylated MLH1 promoter; Lynch Lynch syndrome-associated; Lynch-like Lynch-like syndrome-associated. Asterisk (*) indicates significant difference in mutational prevalence (Fisher’s exact test, *p < 0.05, ***p <0.001, ****p < 0.0001, unadjusted).

The alteration spectrum of key signaling pathways, however, did not show notable differences between the three subgroups (**Figure 6**).

## Discussion

In this study, we performed a comprehensive molecular study of a retrospective EC cohort with 74 dMMR and 43 pMMR patients. Our work builds upon the seminal TCGA classification of endometrial cancer (3), which first established the significance of dMMR as a distinct molecular subtype. Using targeted panel sequencing for 1021 genes, we analyzed gene mutation frequency and genetic mutual relationship of ten canonical cancer signaling pathways, namely Cell Cycle, Hippo, MYC, NOTCH, NRF2, PI3Kinase/Akt, RTK-RAS, TGF-β, TP53, and β-catenin/WNT signaling. DDR-related pathway alterations and TMB levels were also evaluated. According to MMR gene germline mutations and *MLH1* promoter methylation status, “Lynch”, “Lynch-like” and “*MLH1* ^me+^” subgroups of dMMR ECs were identified and compared. We profiled the landscape of gene mutations and key pathway alterations in dMMR ECs, delineated patterns of co-occurrence and mutual exclusivity, and further explored the molecular heterogeneity of dMMR ECs.

Although TCGA established the significance of the dMMR/MSI-H subgroup (3), few cohorts have deeply dissected the genomic profiles of these etiologic subgroups. Our results reveal that these subgroups are not genomically uniform. The striking enrichment of *KEAP1* and *FBXW7* mutations specifically in Lynch-associated cases, based on the number of mutation events, suggests distinct pathogenic processes compared to *MLH1* ^me+^ cases, potentially underlying the variable clinical behaviors and treatment responses observed among historically unified MMR-deficient cancers (38). Soumerai et al. also identified a spectrum of outcomes and actionability within their advanced EC cohort, though their analysis was not focused on differentiating dMMR etiologies (38). By integrating with the TCGA framework and findings from advanced disease cohorts, our work adds resolution to the EC molecular map, strongly suggesting that future prognostic and predictive models should consider both the presence of MMR deficiency and its root cause.

To illustrate the general genetic feature of dMMR ECs, we first compare the mutation profile between dMMR and pMMR group. In consistent with previous reports, the mutation frequencies of *PTEN* and *KRAS*, the two key driver genes of EC carcinogenesis, were significantly higher in dMMR group compared to pMMR group. Also, we found that *ARID1A*, which encodes the subunit of switch/sucrose non-fermentable (SWI/SNF) complex involved in the chromatin remodeling process, was remarkably mutated in our dMMR group. The correlation of increased *ARID1A* mutation frequency and dMMR phenotype has been described extensively in various tumor types (39). However, unlike previous studies, which suggested loss of *ARID1A* expression was more prevalent in sporadic dMMR tumors, we found that *ARID1A* mutation was not correlated with the *MLH1* ^me+^ phenotype in our cohort. Although some *in vivo* studies suggested that *ARID1A* downregulation might mediate modest rather than global DNA methylation regulation early in tumorigenesis (40, 41), it is not completely clear if *ARID1A* mutation is the result or the cause of MMR deficiency secondary to promoter hypermethylation, especially in ECs. Moreover, the *ARID1A* mutations in all three dMMR subgroups were predominantly frameshift events affecting tandem repeat sequences, indicating that they might represent mutational targets of MSI in ECs regardless of the mechanisms underlying MMR deficiency.

On pathway-level, dMMR ECs had significantly higher alteration frequencies in WNT, NOTCH, and Cell Cycle pathway compared to pMMR ECs. The genetic alterations within PI3K and RTK-RAS pathway, the two most commonly altered signaling pathways in ECs, however, did not show remarkable differences between the dMMR and pMMR groups of ECs. Notably, defections in DDR pathways other than MMR, particularly the HRR pathway, were found to be common among our dMMR cases. In a pan-cancer analysis of co-mutations among DDR pathways, the co-existence of HRR and MMR aberrations was associated with higher TMB levels, increased tumor neoantigen load, and upregulated immune gene expression, and considered as a potential biomarker for ICB therapy in some types of non-gynecologic tumors (42). In ECs, co-mutations in the DDR pathway warrant more thorough exploration, in the hope of developing new immunotherapy predictors. The mutual relationships of ten canonical pathways displayed remarkable differences between the dMMR and pMMR ECs. We observed multiple co-existent canonical signaling pathways in the pMMR group, rather than the dMMR group, which indicates a potential for combination therapy in pMMR ECs.

We further explored the interactions within pathways both in dMMR and pMMR ECs, which have not been addressed in previous studies. The most significant difference was reflected in the WNT signaling pathway. Among ECs displaying genetic alterations in WNT pathway components, we found that *CTNNB1* mutations, generally considered as the hallmark of aberrant Wnt/β-catenin signaling, were completely absent in the dMMR group while presented in half of the pMMR group. Consistent with our results, Byron et al. (43) also found that *CTNNB1* mutations were significantly less common in microsatellite instability (MSI) ECs. However, there was still a small proportion of MSI ECs with *CTNNB1* mutations in their cohort. This discrepancy may be due to our relatively small sample size and racial difference. Additionally, frequently heterogeneous and subclonal *CTNNB1* status has been observed in ECs (44), which might also contribute to the discrepancy. In addition, several prior studies revealed the association of *CTNNB1* mutation with increased risk of recurrence in ECs but generally included a heterogeneous population comprised of both dMMR and pMMR cases (45, 46). Our data suggested that it is needed to evaluate the prognostic value of *CTNNB1* mutation in separately in both dMMR and pMMR groups. Moreover, the interaction relationship among other key WNT signaling pathway genes displayed noticeable differences between dMMR and pMMR groups, manifesting as mutually exclusive relationships in dMMR group, and co-occurrence relationships in pMMR group. This finding indicated the different modes of WNT signaling pathway aberrations between dMMR and pMMR ECs, suggesting that alternative mechanisms might be responsible for WNT pathway activation in EC tumorigenesis. In most cases, PI3K signaling aberrations were caused by *PTEN* mutations accompanied by *PIK3CA* and/or *PIK3R1* mutations, verifying the synergistic effects of PI3K pathway mutations in EC tumorigenesis proposed by previous studies (47, 48). *KRAS* mutation was shown to be the primary mechanism of RTK-RAS signaling activation. Whilst canonical *KRAS* mutations do not co-exist with other alterations within the RTK-RAS pathway, non-canonical *KRAS* mutations generally co-occur with *NF1* dysfunctional mutations, suggesting that such pairs of mutations might act cooperatively to provide a selective advantage in tumorigenesis of ECs regardless of MMR status (49).

It has been well-established that dMMR status is a favorable prognostic factor in certain cancer types and a predictor for anti-PD-1/PD-L1 immunotherapy efficacy in solid tumors. However, previous studies reported inconsistent findings comparing outcomes between dMMR ECs and ECs of non-specific molecular profile (NSMP) (4, 50, 51). It has also been reported that ECs harboring *MLH1* hypermethylation showed poorer response to ICB therapy compared with Lynch syndrome-associated ECs, which implied the different immunotherapy response-associated genetic alternations may exist in etiologically distinct EC subgroups.

The Lynch-associated ECs in our cohort were enriched for *FBXW7* and *KEAP1* mutations. This observed enrichment suggests a potential association of *FBXW7* and/or *KEAP1* mutations with poor response to checkpoint blockade therapy in multiple solid tumors, including ECs, as has been evidenced in clinical studies (52–54). In recent published *in vivo* studies, *FBXW7* and *KEAP1* were suggested to confer immune checkpoint blockade by alternating tumor microenvironment instead of directly modifying the tumor, through the way of decreasing T-cell infiltration and downregulating IFN-γ signaling, respectively (55, 56). However, given the small sample size of our Lynch subgroup, these findings should be interpreted cautiously and require validation in larger cohorts.

The study has several potential limitations. Firstly, our study is limited by its retrospective, single-center design and the imbalance in cohort size and stage distribution between groups. Although the pMMR group contained a higher proportion of advanced-stage (III-IV) disease, our stratified analyses by stage (I-II *vs*. III-IV) robustly demonstrated that the core molecular differences (TMB, pathway alterations) between dMMR and pMMR groups were maintained within both stage categories. This strengthens the conclusion that these differences are driven primarily by MMR status. Nonetheless, future prospective, multi-center studies are warranted to validate these findings. Besides, the analyses of etiologic dMMR subgroups are exploratory in nature due to the small sample sizes of these subgroups (particularly the Lynch subgroup with only 13 cases) and require validation in larger studies. Secondly, regarding the statistical analyses, while we have specified the use of Fisher’s exact tests for categorical comparisons as appropriate, we acknowledge that no corrections for multiple comparisons were applied to the p-values in the exploratory analyses of dMMR subgroups. This was primarily due to the exploratory and hypothesis-generating nature of these subgroup analysis and the concern that overly stringent correction methods might obscure potentially important biological findings in this context. However, we recognize that this increases the risk of Type I errors, and therefore, the statistical significances reported in these subgroup comparisons should be interpreted with caution and require validation in future studies with larger sample sizes. Thirdly, a key limitation of our study is the constraint imposed by the targeted gene panel. For instance, our analysis of the NHEJ pathway was limited to only two genes (*PRKDC* and *RAD50*), as core components like *XRCC4*, *XRCC5*, *XRCC6*, and *LIG4* were not included. Therefore, the alteration frequency we report for NHEJ is likely an underestimate, and our findings regarding this pathway are not conclusive. Future studies employing whole-exome sequencing or custom DDR-focused panels will be essential to capture the complete mutational landscape and functional interplay within these critical pathways. Finally, the follow-up period in this study was relatively short, and the majority of patients did not experience disease-related endpoint events. For example, although we observed a numerical reduction in recurrence and mortality rates in the high-TMB pMMR group (all of which were *POLE*mut) (22.2% *vs*. 44.1%; 0% *vs*. 5.9%), this trend did not reach statistical significance (p = 0.363). This is likely attributable to the limited sample size of the *POLE*mut subgroup and its low event rate, which constrained the statistical power. Therefore, extended follow-up is warranted in the future to capture long-term outcomes. Nonetheless, our findings align with established literature indicating that *POLE*mut tumors confer an excellent prognosis. Therefore, identifying high-TMB pMMR tumors and further confirming *POLE* status is critical for accurate prognostic stratification. Relying solely on TMB values without investigating the underlying driver (such as *POLE* status) may obscure this robust prognostic signal.

To summarize, our comprehensive molecular study uncovered significant differences in the mutation spectrum and interaction patterns of key genes and pathways between dMMR and pMMR ECs. We also revealed the molecular heterogeneity among dMMR subgroups with different etiologies. Our findings may have potential prognostic and therapeutic implications.

## Data Availability

The datasets presented in this study can be found in online repositories. The names of the repository/repositories and accession number(s) can be found in the article/**Supplementary Material**.

## Supplementary material

Supplementary Figure 1 Representative IHC staining patterns of the four MMR proteins in endometrial carcinoma. Intact or lost nuclear expression of MLH1, PMS2, MSH2, and MSH6 proteins. The loss of nuclear staining in tumor cells is assessed against internal positive control cells (e.g., stromal cells, lymphocytes). IHC immunohistochemical, MMR mismatch repair, dMMR deficient MMR.

Supplementary Figure 2 Quality control metrics distribution across 117 Samples. Avg Depth Average sequencing depth on target without duplicated reads, Median Depth Median sequencing depth on target without duplicated reads, Coverage@100x (%): Fraction of target covered with >= 100x, Coverage@10x (%) Fraction of target covered with >= 10x, Capture Efficiency (%): Fraction of effective bases on target (capture efficiency; without duplicated reads), Mapping Rate Fraction of reads mapped to genome.

Supplementary Figure 3 Survival outcomes by MMR status. (a) progression-free survival (PFS). (b) overall survival (OS). MMR mismatch repair, dMMR deficient mismatch repair, pMMR proficient mismatch repair.

Supplementary Figure 4 Survival outcomes between nine high-TMB pMMR patients and the remaining pMMR patients. (a) progression-free survival (PFS). (b) overall survival (OS). TMB: tumor mutational burden, pMMR proficient mismatch repair.

Supplementary Figure 5 Tumor mutational burden (TMB) by MMR Status in (a) early-stage (I/II) and (b) late-stage (III/IV) Cohorts. MMR mismatch repairMuts/Mb mutations per megabase. Asterisk(*) significant difference in mutational prevalence (Fisher’s exact test, **p < 0.01, ****p < 0.0001, FDR-corrected).

Supplementary Figure 6 Distribution of WNT, RTK-RAS, PI3K, TGF-β, Cell Cycle, Hippo, NRF2, NOTCH, MYC and TP53 pathways and DDR pathways with respect to their mutation frequency by MMR status in Early-Stage (I/II) and Late-Stage (III/IV) Cohorts. (a, c) Distribution of WNT, RTK-RAS, PI3K, TGF-β, Cell Cycle, Hippo, NRF2, NOTCH, MYC and TP53 pathways in early-stage and late-stage cohorts respectively. (b, d) Distribution of DDR pathways in early-stage and late-stage cohorts respectively; dMMR deficient mismatch repair, pMMR proficient mismatch repair, EC endometrial carcinoma, DDR DNA damage response, MMR mismatch repair, HRR homologous recombination repair, FA Fanconi anemia, BER base excision repair, CPFs checkpoint factors, NER nucleotide excision repair, NHEJ nonhomologous end joining, and TLS translesion synthesis. Asterisk (*) significant difference in mutational prevalence (Fisher’s exact test, *p < 0.05, **p < 0.01, FDR-corrected).

Supplementary Figure 7 Progression-free survival (PFS) between 3 dMMR subgroups. MLH1me+ hypermethylated *MLH1* promoter, Lynch Lynch syndrome-associated, Lynch-like Lynch-like syndrome-associated, dMMR deficient mismatch repair.
