# Supplementary figures and images for: Mutation profile and molecular heterogeneity in mismatch repair deficient endometrial carcinoma
Source: Front Oncol. 2025 Oct 21;15:1596879. doi: 10.3389/fonc.2025.1596879 (PMC12583210; doi:10.3389/fonc.2025.1596879)

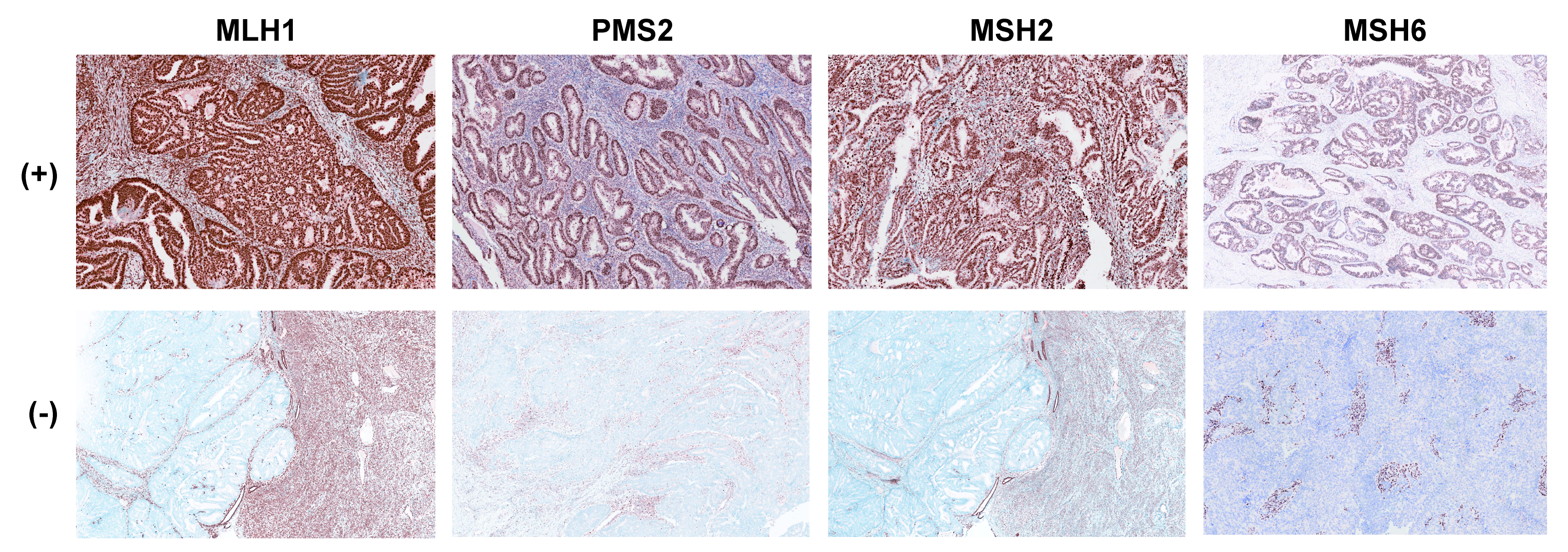

Supplement: Supplementary Figure 1 — Representative IHC staining patterns of the four MMR proteins in endometrial carcinoma. Intact or lost nuclear expression of MLH1, PMS2, MSH2, and MSH6 proteins. The loss of nuclear staining in tumor cells is assessed against internal positive control cells (e.g., stromal cells, lymphocytes). IHC immunohistochemical, MMR mismatch repair, dMMR deficient MMR. [file Image1.tif]

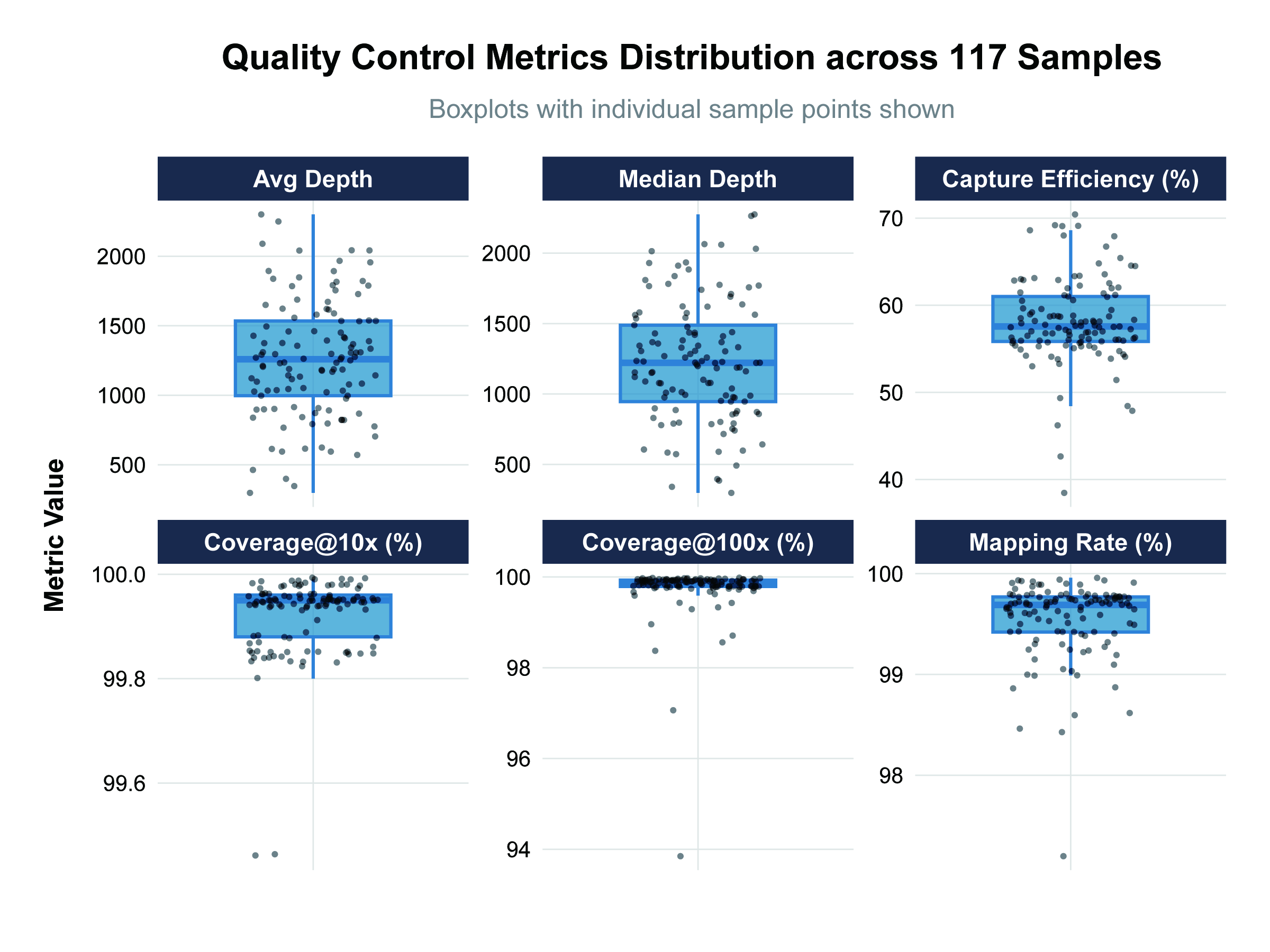

Supplement: Supplementary Figure 2 — Quality control metrics distribution across 117 Samples. Avg Depth Average sequencing depth on target without duplicated reads, Median Depth Median sequencing depth on target without duplicated reads, Coverage@100x (%): Fraction of target covered with >= 100x, Coverage@10x (%) Fraction of target covered with >= 10x, Capture Efficiency (%): Fraction of effective bases on target (capture efficiency; without duplicated reads), Mapping Rate Fraction of reads mapped to genome. [file Image2.tif]

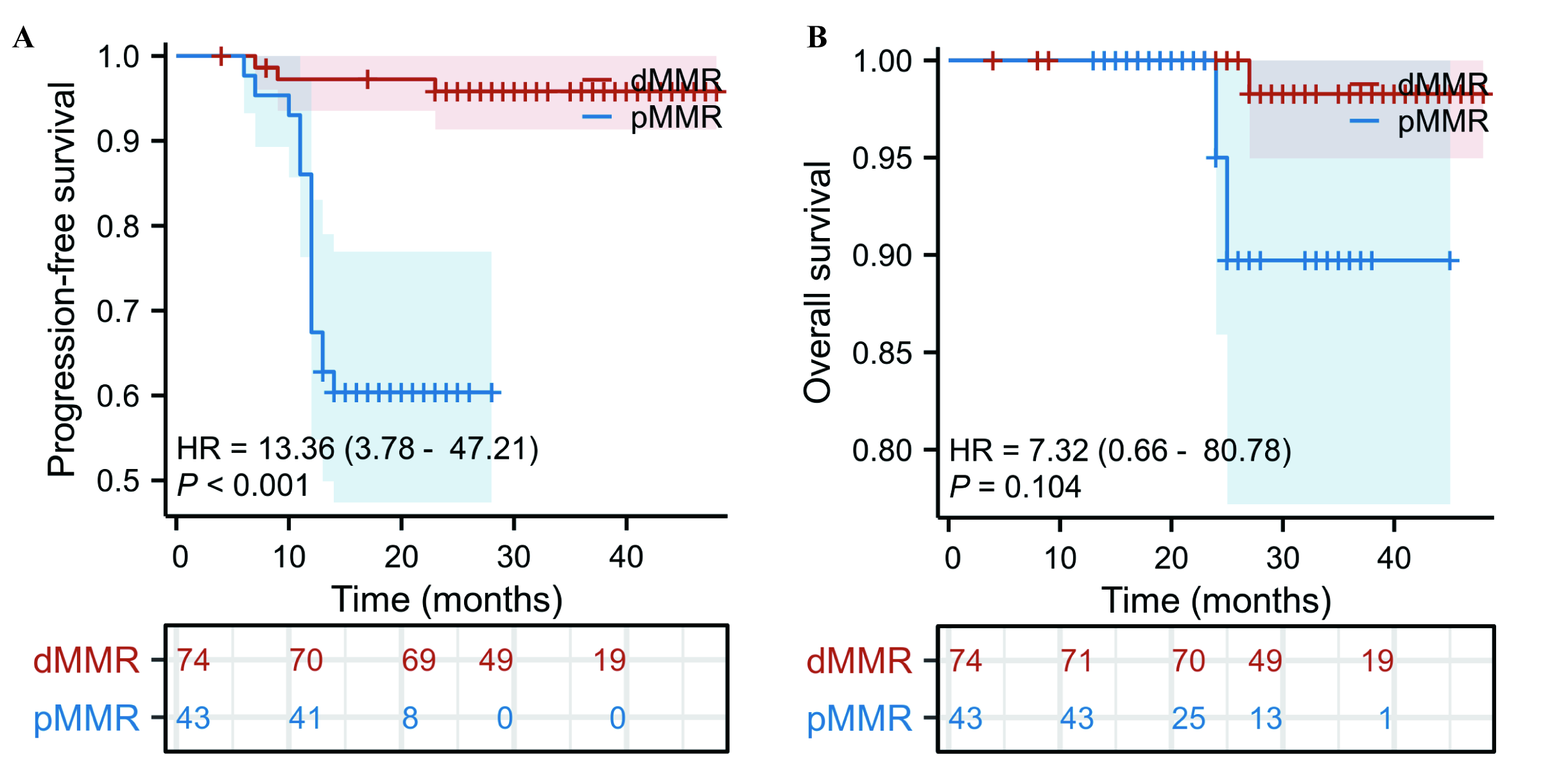

Supplement: Supplementary Figure 3 — Survival outcomes by MMR status. (a) progression-free survival (PFS). (b) overall survival (OS). MMR mismatch repair, dMMR deficient mismatch repair, pMMR proficient mismatch repair. [file Image3.tif]

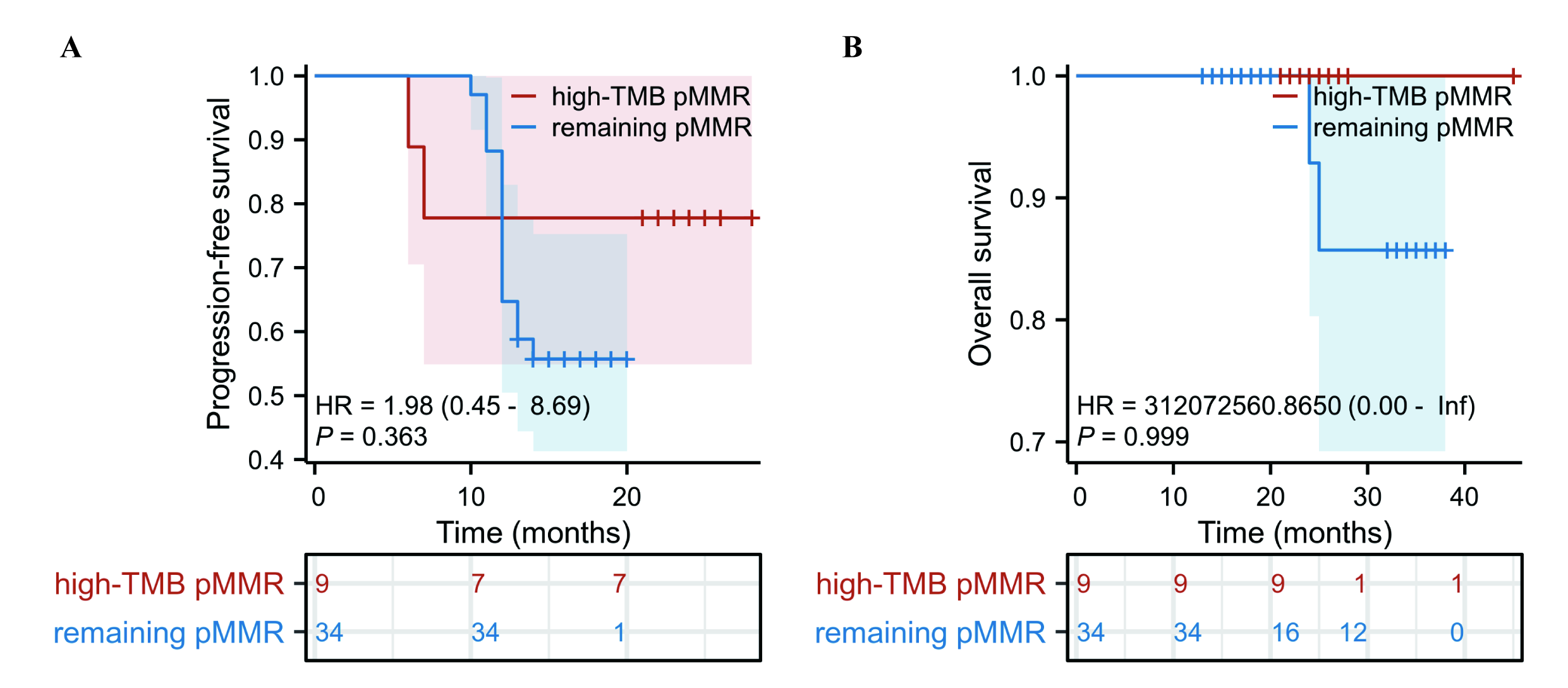

Supplement: Supplementary Figure 4 — Survival outcomes between nine high-TMB pMMR patients and the remaining pMMR patients. (a) progression-free survival (PFS). (b) overall survival (OS). TMB: tumor mutational burden, pMMR proficient mismatch repair. [file Image4.tif]

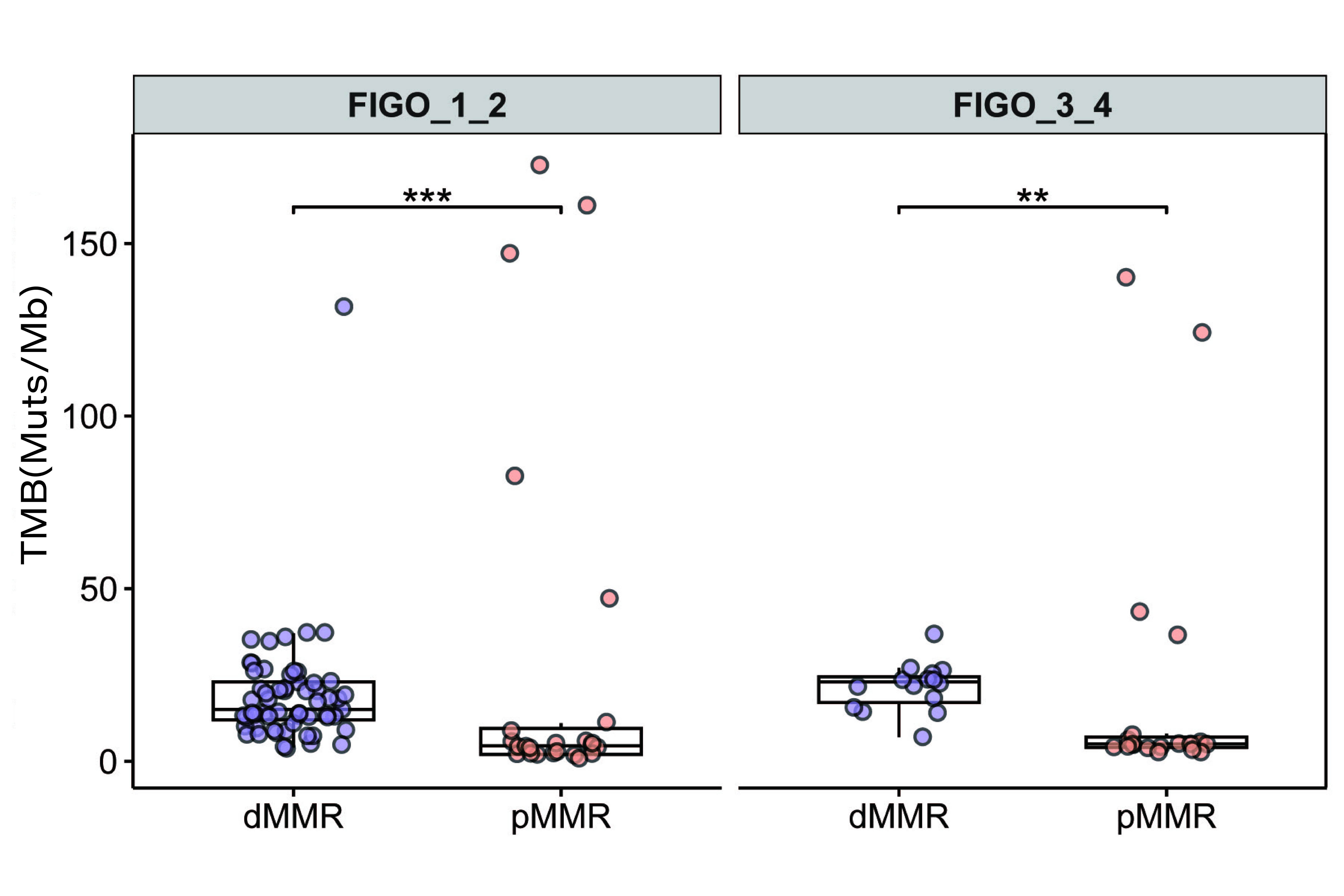

Supplement: Supplementary Figure 5 — Tumor mutational burden (TMB) by MMR Status in (a) early-stage (I/II) and (b) late-stage (III/IV) Cohorts. MMR mismatch repairMuts/Mb mutations per megabase. Asterisk(*) significant difference in mutational prevalence (Fisher’s exact test, **p < 0.01, ****p < 0.0001, FDR-corrected). [file Image5.jpg]

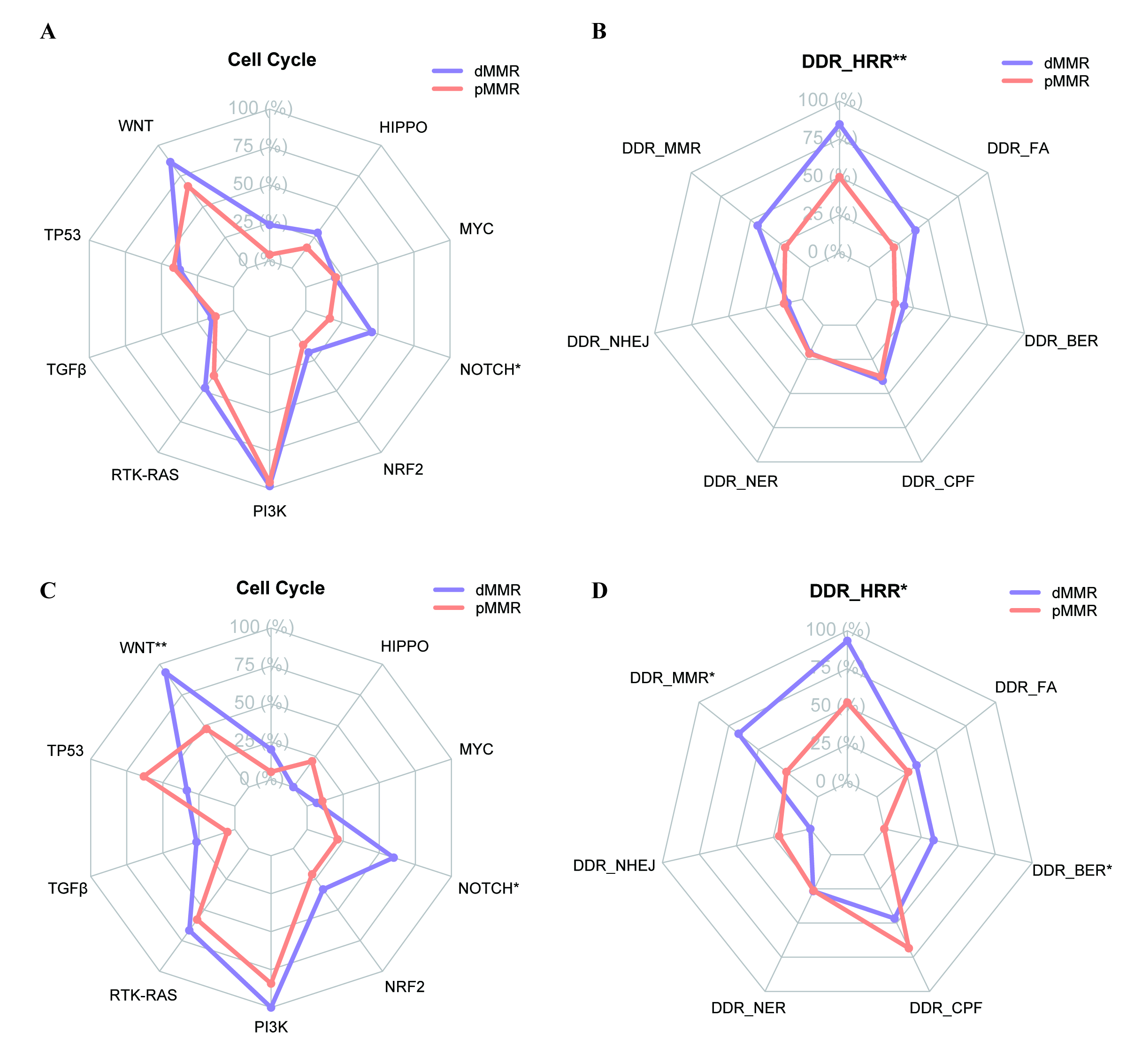

Supplement: Supplementary Figure 6 — Distribution of WNT, RTK-RAS, PI3K, TGF-β, Cell Cycle, Hippo, NRF2, NOTCH, MYC and TP53 pathways and DDR pathways with respect to their mutation frequency by MMR status in Early-Stage (I/II) and Late-Stage (III/IV) Cohorts. (a, c) Distribution of WNT, RTK-RAS, PI3K, TGF-β, Cell Cycle, Hippo, NRF2, NOTCH, MYC and TP53 pathways in early-stage and late-stage cohorts respectively. (b, d) Distribution of DDR pathways in early-stage and late-stage cohorts respectively; dMMR deficient mismatch repair, pMMR proficient mismatch repair, EC endometrial carcinoma, DDR DNA damage response, MMR mismatch repair, HRR homologous recombination repair, FA Fanconi anemia, BER base excision repair, CPFs checkpoint factors, NER nucleotide excision repair, NHEJ nonhomologous end joining, and TLS translesion synthesis. Asterisk (*) significant difference in mutational prevalence (Fisher’s exact test, *p < 0.05, **p < 0.01, FDR-corrected). [file Image6.tif]

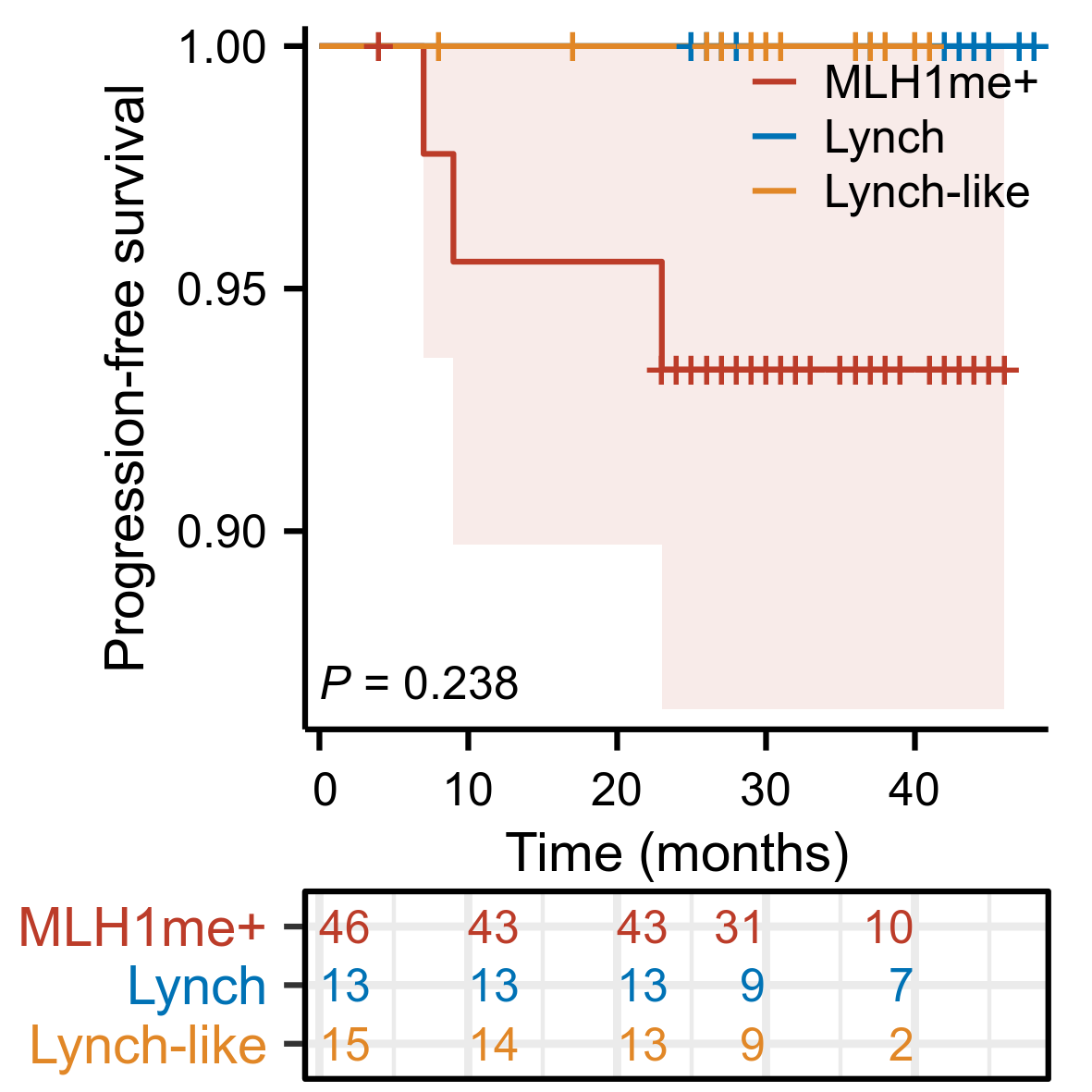

Supplement: Supplementary Figure 7 — Progression-free survival (PFS) between 3 dMMR subgroups. MLH1me+ hypermethylated MLH1 promoter, Lynch Lynch syndrome-associated, Lynch-like Lynch-like syndrome-associated, dMMR deficient mismatch repair. [file Image7.tiff]
