# Supplementary material for: Mutation profile and molecular heterogeneity in mismatch repair deficient endometrial carcinoma
Source: Front Oncol. 2025 Oct 21;15:1596879. doi: 10.3389/fonc.2025.1596879 (PMC12583210; doi:10.3389/fonc.2025.1596879)
Supplement: Supplementary file 10 [file Table3.docx]

| **Supplementary Table 3 Clinicopathological Features of the High-TMB Subgroup Compared to Other pMMR Endometrial Carcinomas** | | | |
| --- | --- | --- | --- |
| **Clinicopathologic charactristics** | **TMB-H(n=9)** | **other(n=34)** | **P** |
| **Age** |  |  | 0.332 |
| <60 | 6 (66.75) | 29 (85.3%) |  |
| ≥60 | 3 (33.3%) | 5 (14.7%) |  |
| **FIGO stage** |  |  | 0.292 |
| I | 5 (55.6%) | 10 (29.4%) |  |
| II | 0 (0%) | 9 (26.5%) |  |
| III | 2 (22.2%) | 6 (66.7%) |  |
| IV | 2 (22.2%) | 9 (26.5%) |  |
| **Tumor grade/histology** |  |  | 0.277 |
| Endometrioid | 9 (100%) | 32 (94.1%) |  |
| Grade1 | 0 (0%) | 9 (26.5%) |  |
| Grade2 | 5 (55.6%) | 14 (41.2%) |  |
| Grade3 | 4 (44.4%) | 9 (26.5%) |  |
| Non-endometrioid | 0 (0%) | 2 (5.9%) |  |
| **Surgical procedure** |  |  | 1 |
| Laparoscopic | 7 (77.8%) | 28 (82.4%) |  |
| Laparotomic | 2 (22.2%) | 6 (17.6%) |  |
| **Lymphadenectomy** |  |  | 0.365 |
| Sentinel node biopsy | 4 (44.4%) | 21 (61.8%) |  |
| Systemic lymph node dissection | 2 (22.2%) | 3 (8.8%) |  |
| Not done | 3 (33.3%) | 10 (29.1%) |  |
| **Median follow-up (range), months** | 22 month(13-45） |  |  |
| **Recurrence/Metastasis** | 2 (22.2%) | 15 (44.1%) | PFS=0.363 |
| **Death** | 0 (0%) | 2 (5.9%) | OS=0.999 |

| **Supplementary Table 4 Clinicopathological Features of the dMMR subgroups** | | | | |
| --- | --- | --- | --- | --- |
| **Clinicopathologic charactristics** | **MLH1me+(n=46)** | **Lynch(n=13)** | **Lynch-like(n=15)** | **P** |
| **Age** |  |  |  | 0.138 |
| <60 | 31 (67.4%) | 12 (92.3%) | 9 (60%) |  |
| ≥60 | 15 (32.6%) | 1 (8.3%) | 6 (40%) |  |
| **FIGO stage** |  |  |  | 0.279 |
| I | 35 (76.1%) | 9 (69.2%) | 13 (86.7%) |  |
| II | 0 (0%) | 1 (7.7%) | 1 (6.7%) |  |
| III | 10 (21.7%) | 3 (23.1%) | 1 (6.7%) |  |
| IV | 1 (2.2%) | 0 (0%) | 0 (0%) |  |
| **Tumor grade/histology** |  |  |  | 0.513 |
| Endometrioid | 46 (100%) | 13 (100%) | 15 (100%) |  |
| Grade1 | 15 (32.6%) | 7 (53.8%) | 7 (46.7%) |  |
| Grade2 | 24 (52.2%) | 4 (30.8%) | 5 (33.3%) |  |
| Grade3 | 7 (15.2%) | 2 (15.4%) | 3 (20%) |  |
| Non-endometrioid | 0 (0%) | 0 (0%) | 0 (0%) |  |
| **Surgical procedure** |  |  |  | 0.664 |
| Laparoscopic | 42 (91.3%) | 12 (92.3%) | 15 (100%) |  |
| Laparotomic | 4 (8.7%) | 1 (7.7%) | 0 (0%) |  |
| **Lymphadenectomy** |  |  |  | 0.544 |
| Sentinel node biopsy | 9 (19.6%) | 5 (38.5%) | 4 (26.7%) |  |
| Systemic lymph node dissection | 4 (8.7%) | 0 (0%) | 0 (0%) |  |
| Not done | 33 (71.7%) | 8 (61.5%) | 11 (73.3%) |  |
| **Median follow-up (range), months** | 35month(4-48） |  |  |  |
| **Recurrence/Metastasis** | 3 (6.5%) | 0 (0%) | 0 (0%) | PFS=0.238 |
| **Death** | 1 (2.2%) | 0 (0%) | 0 (0%) | OS=1 |
